# *Candida tropicalis* spondylodiskitis in a patient with carcinoma of sigmoid colon: a case report

**DOI:** 10.1186/1757-1626-1-66

**Published:** 2008-07-29

**Authors:** Chandra JKB Parthiban, Shiju A Majeed, Shanthanam S Mahalingam

**Affiliations:** 1 Division of Spine Surgery, Kovai Medical Center and Hospital, Coimbatore, India

## Abstract

Fungal spondylodiskitis is being increasingly reported in immunocompromised patients. A patient who presented with severe back pain three months following laparoscopic resection of Carcinoma of Sigmoid colon is presented here. Magnetic Resonance Imaging of the spine showed evidence of multilevel lumbar spondylodiskitis. Biopsy done via the posterior approach showed Candida tropicalis sensitive to fluconazole and resistant to Amphotericin B. Patient was treated with fluconazole and had good relief. Posterior pedicle screw stabilization was done to provide mechanical stability.

## Background

Vertebral osteomyelitis and diskitis form part of a spectrum of infections of the vertebral column. Infections of the vertebral column can be due to pyogenic, mycobacterial or fungal organisms. Fungal spondylodiskitis are commonly seen in HIV patients, Intravenous drug abusers, steroid users, bone marrow transplant recipients, renal transplant recipients and in patients with other immune incompetencies. Few case reports of fungal vertebral osteomyelitis in patients with history of fungemia have been published. The presence of a malignancy is also a risk factor in the development of fungal vertebral infections.

## Case Report

A 62 year old man presented to our clinic with history of severe low backache over the last 3 months. He gives history of being treated for Early Carcinoma of Sigmoid colon immediately prior to the onset of backache. He had underwent laparoscopic resection of the tumour and subsequent anastomosis of the large intestine. The histopathology showed good tumour clearance. Initial workup for metastasis was negative. He developed anastomotic leak and an enterocutaneous fistula and was reoperated via a laparotomy. Defunctioning colostomy was done. When he presented to us, he had a colostomy bag and his sinus had healed. The backpain was intense when he attempted to move about. Resting in bed gave him some relief. His examination was negative except for paraspinal muscle spasm He had no neurological deficit.

**Figure 1:**
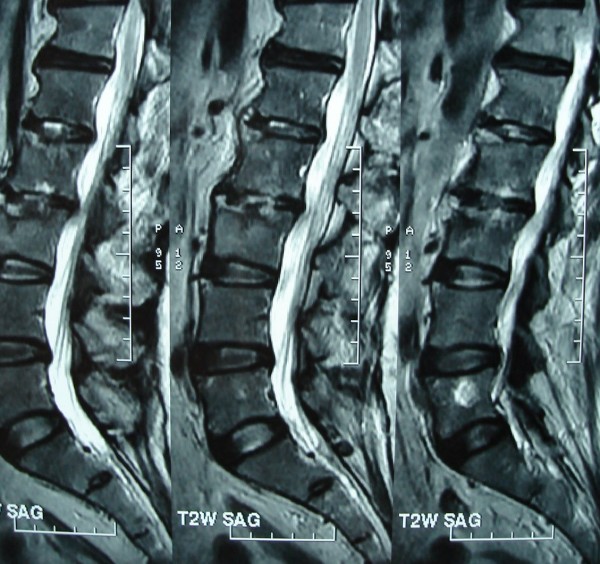
sagittal T1 MRI showing diskitis and vertebral signal changes suggestive of osteomyelitis

**Figure 2:**
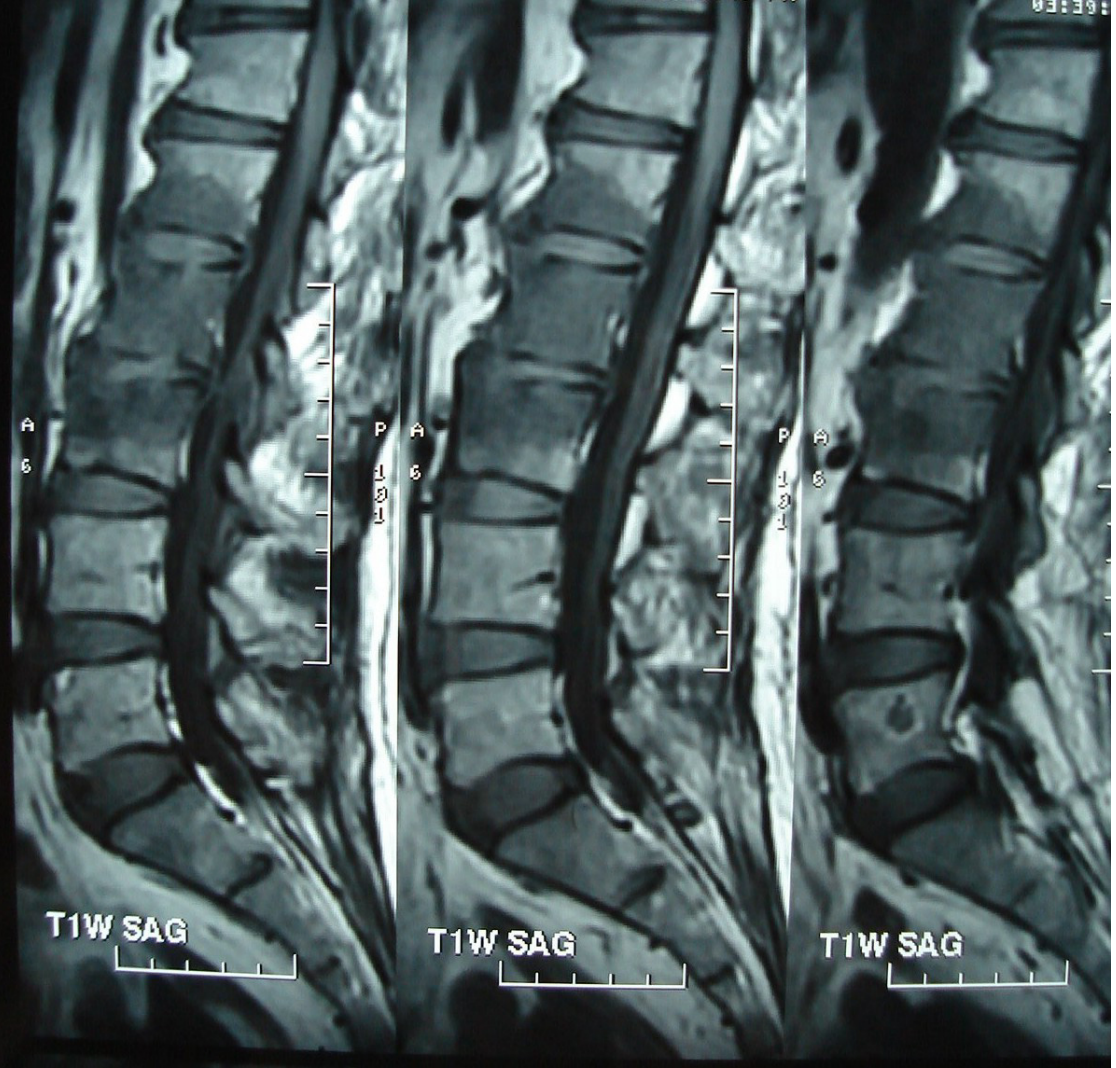
sagittal T2 MRI showing diskitis and vertebral signal changes suggestive of osteomyelitis

**Figure 3:**
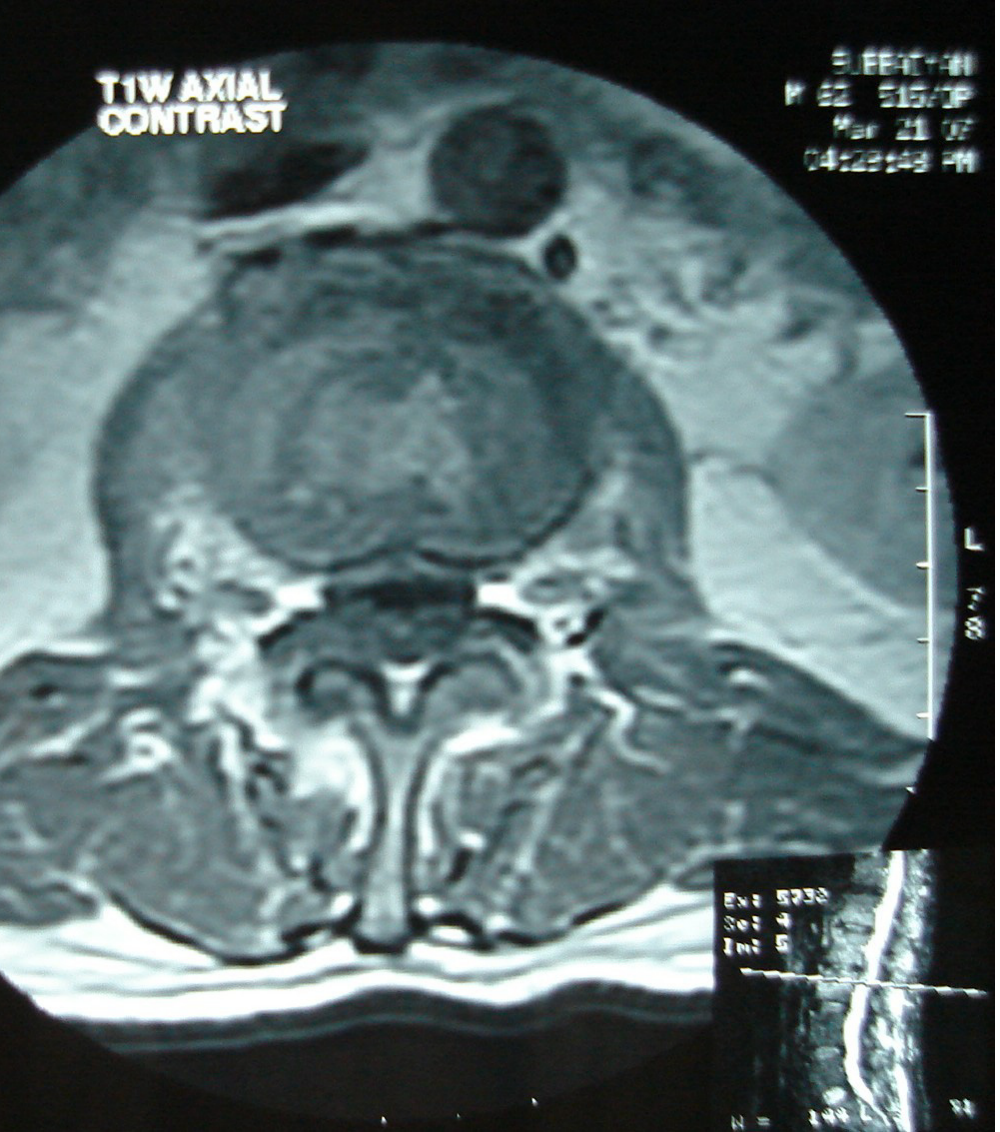
Axial T1 contrast showing the above lesions

**Figure 4:**
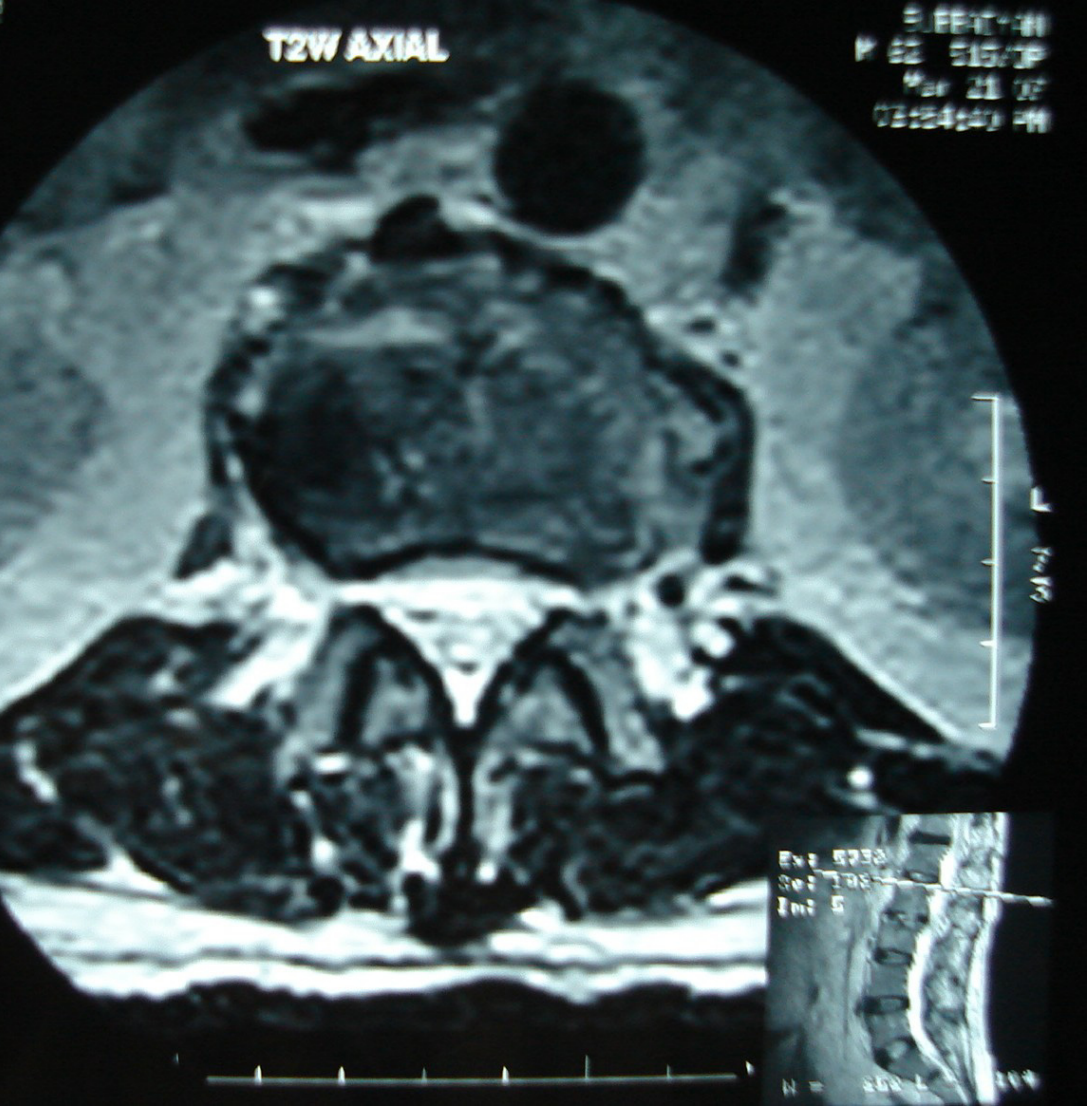
Axial T2 MRI images showing the above lesions

His Erythrocyte Sedimentation rate was 116 mm/first hour. Radiographs of the Lumbosacral spine showed sclerosis of the L2,3 endplates. MRI showed spondylodiskitis of L2,3 vertebrae and L1,2 vertebrae(Figs. 1,2,3,4). The involvement of L2,3 was more with early changes in L1,2 disc space. The patient had been on long duration of antibiotics following the abdominal surgery.

Considering the MRI picture, an anterior debridement and biopsy via a retroperitoneal approach was thought of. But due to the colostomy wound and the laparotomy wound, the chance of contaminating the surgical wound was high. Also the possibility of peritoneal adhesions make anterior dissection difficult. Hence the lesion was approached via a posterior route. L2 laminectomy was performed, L2,3 disc space curetted out and sent for histopathology and culture. The spine was stabilized from L1 to L4 via pedicle screw instrumentation. Instrumentation in infected spine is now very well recognized as a therapeutic option to provide mechanical stability [1,2].

Cultures for bacteria including mycobacterium were negative. Myco 3 PCR for Tuberculosis was negative. Fungal Culture grown on Saborauds Tween 80 corn meal agar showed yeast cells of Candida tropicalis. The organism was sensitive to Fluconazole and resistant to Amphotericin B.

Patient was started on Fluconazole – 400 mg intravenously for two weeks and then orally for a period of 10 weeks. Patient was gradually mobilized. His ESR dropped 40 mm/first hour and he had good symptomatic relief. He later underwent colostomy closure uneventfully and is back to normal activities.

## Discussion

Candida spondylodiskitis is a very rare entity. A few cases of C. albicans causing diskitis have been reported[3]. C. tropicalis diskitis has not been reported.

Candida tropicalis has white to cream coloured, smooth glabrous and yeast like appearance in Sabourauds dextrose agar[4]. Under microscope, they have spherical or subspherical yeast like cells or blastoconidia measuring 3.0 – 5.5 × 4.0 – 9.0 micrometre size. In cornmeal and Tween 80 agar, C. tropicalis has abundant, long, wavy, branched pseudohyphae with numerous ovoid blastoconidia. They have no terminal vesicles (chlamydoconidia)

A comparison of C. tropicalis with the more common C. albicans is summarized in Table 1.

**Table 1:** showing the differences between C. albicans and C. tropicalis[2].

**Property**	**C. tropicalis**	**C. albicans**
Presence of chlamydoconidia	No	Yes
Germ tube test	Negative	Positive with 3 hrs
Hydrolysis of urea	Negative	Negative
Growth on cycloheximide medium	Positive	Positive
Growth at 37 degree Celsius	Positive	Positive

## Conclusion

To our best of knowledge, Candida tropicalis spondylodiskitis in a patient with Carcinoma of colon has not been reported. Early diagnosis of fungal osteomyelitis can help one initiate appropriate antifungal therapy. This is found to halt the progression of the disease and its neurological sequelae. This may obviate the need for surgery also. Should the need for surgery arise, anterior debridement and grafting happens to be the procedure of choice as advised by many authors[1-3]. However, the previous laparotomy and presence of colostomy bag needs one to modify the surgical approach in such patients. Posterior approach reduces the morbidity and also gives sufficient material to yield a diagnosis. Mechanical stability can be regained easily.

**Figure 5:**
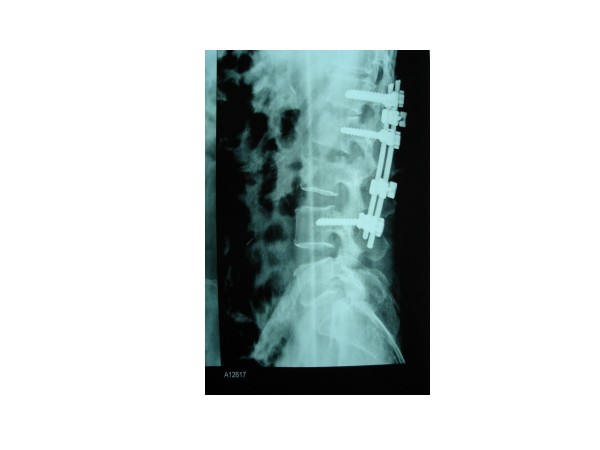
postoperative radiograph of posterior pedicle screw instrumentation

**Figure 6:**
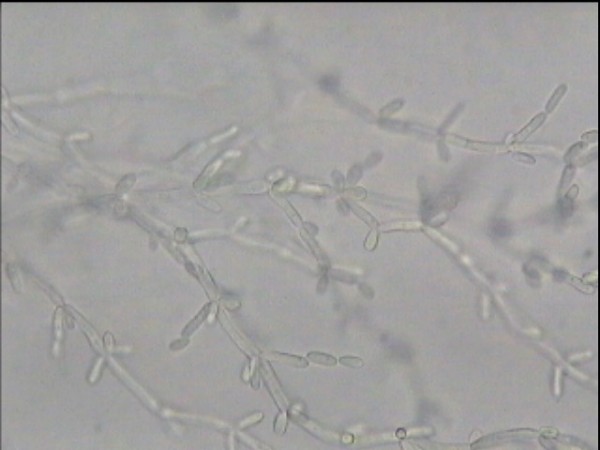
photomicrograph of *C. tropicalis*

**Figure 7:**
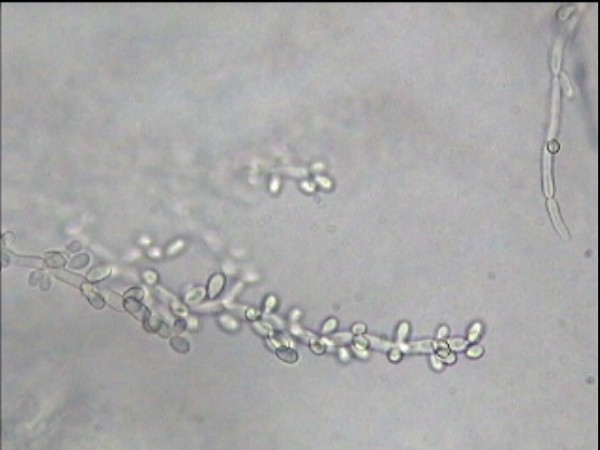
photomicrograph of *C. tropicalis* at a higher magnification

## Abbreviations

MRI: Magnetic Resonance Imaging; ESR: Erythrocyte Sedimentation Rate.

## Competing interests

The authors declare that they have no competing interests.

## Authors' contributions

The first and second author were involved in the treatment of the above case as well as in the preparation of the manuscript. The third author did the literature review. All three have read and approved the final manuscript.

## Consent

Written informed consent was obtained from the patient for publication of this case report and any accompanying images. A copy of the written consent is available for review by the Editor-in-Chief of this journal.
